# The interaction between arterial oxygenation and carbon dioxide and hospital mortality following out of hospital cardiac arrest: a cohort study—do not dismiss confounders!

**DOI:** 10.1186/s13054-020-03196-8

**Published:** 2020-09-03

**Authors:** Romain Jouffroy, Benoît Vivien

**Affiliations:** SAMU de Paris, Service d’Anesthésie Réanimation, Hôpital Universitaire Necker - Enfants Malades, Assistance Publique - Hôpitaux de Paris, APHP. Centre and Université de Paris, Paris, France

To the Editor,

In a recent study, McGuigan et al. [1] reported that a low PaO2/FiO2 ratio, hypoxemia, and hypocapnia are associated with higher mortality following out of hospital cardiac arrest (OHCA) and secondly that PaCO2 modifies the relationship between oxygenation and mortality following OHCA.

Whereas the authors should be congratulated for their noteworthy study, we believe that their interpretation requires some cautions.

First, McGuigan et al. choose a PaO2 > 100 mmHg to define hyperoxemia and a PaO2 of 150–200 mmHg as reference category. Conversely, in the two first princeps papers reporting an association between arterial hyperoxia following resuscitation from cardiac arrest and in-hospital mortality, Kilgannon et al. [2] and Bellomo et al. [3] have defined hyperoxia as a PaO2 > 300 mmHg. Consequently, the large interval of PaO2 considered by McGuigan et al. [1] does not guarantee that mortality is uniform for all the patients presenting with a PaO2 > 100 mmHg, and could fully explain the discrepancy with the results of Kilgannon et al. [2] and Bellomo et al. [3].

Secondly, the interpretation of PaO2 and PaCO2 values would have been more pertinent if the authors had presented the time and cause of death of the patients. On the one hand, hypoxemic patients (PaO2 < 60 mmHg) were probably those suffering from respiratory injuries following cardiac arrest, i.e., mainly aspiration and/or alveolar hemorrhage. While it could be hypothesized that a protective ventilation strategy had been initiated for them, inducing high PaCO2 values, the direct effect of PaCO2 by itself is limited on respiratory function and therefore on mortality from respiratory origin [4]. On the other hand, for patients presenting with neurological injury but without respiratory insufficiency, the effect of PaCO2 should be considered as ambivalent. In the absence of cerebral edema, hypocapnia is deleterious by reducing cerebral blood flow and exacerbating cerebral ischemia; on the opposite, for patients presenting with cerebral edema following cardiac arrest resuscitation, hypercapnia is deleterious by inducing cerebral vasodilation. Nevertheless, the cause of death for patients with neurological assault following cardiac arrest, whatever they were hypocapnic or hypercapnic, was probably mainly from neurological origin.

Finally, the post cardiac arrest resuscitation period must be considered as a bundle of care, in which the therapeutic strategy should be multimodal and mostly individualized for each patient [5].

## Data Availability

Not applicable.
