# Supplementary material for: The interaction between arterial oxygenation and carbon dioxide and hospital mortality following out of hospital cardiac arrest: a cohort study—do not dismiss confounders!
Source: Crit Care. 2020 Sep 3;24:544. doi: 10.1186/s13054-020-03196-8 (PMC7650289; doi:10.1186/s13054-020-03196-8)
Supplement: Related texts

# Reply: Authors’ response

**Authors:** Peter J. McGuigan, Manu Shankar-Hari, David A. Harrison, John G. Laffey, Danny F. McAuley

Dear Editor,

We thank Dr. Jouffroy and Professor Vivien for their interest in our study. We would point out that the reference category in our study was hyperoxia (defined as a PaO2 > 100 mmHg). We apologize if this was not clear in the original text.

Firstly, the authors argue that our choice of a PaO2 > 100 mmHg as a threshold for hyperoxia could explain the differences between our findings and those of Kilgannon et al. and Bellomo et al. [2, 3]. For this reason, we performed a sensitivity analysis examining the effect of varying effects of thresholds of hyperoxia on mortality (presented on the additional file 1). This found no association between a PaO2 > 300 mmHg and mortality.

We agree with the authors that it is important not to miss confounding variables. Importantly, Kilgannon et al. and Bellomo et al. included patients who died within the first 24 h of ICU admission; these patients were excluded from our primary analysis. When they were included in a further sensitivity analysis, those with a PaO2 > 300 mmHg had a higher mortality. This was consistent with the findings of Kilgannon et al. and Bellomo et al. The median ICU length of stay for non-survivors following cardiac arrest is short, typically, 1.5–2 days [2, 3]. Kaplan-Meier analysis demonstrates the majority of excess in mortality associated with hyperoxia occurs in the first day of ICU admission [2]. As discussed in our paper, two thirds of patients who die in the first 24 h have withdrawal of life sustaining therapy based on pre-existing co-morbidities or perceived poor neurological prognosis [6]. Yet, these patients have a disproportionate risk of exposure to hyperoxia [7]. This confounder may, in part, explain the association between hyperoxia with a PaO2 > 300 mmHg and mortality. To remove this confounder, we excluded those who died in the first 24 h of ICU admission. The difference in population studied in our paper may help account for the differences between our findings and previous research [2, 3].

Secondly, we agree with the authors that the mechanism of death would have further aided the interpretation of our findings. We recognize this is a limitation of our research.

Finally, while we agree that bundles of care are relevant in the management of post cardiac arrest patients [5], further research is needed to inform mandatory components of these care bundles. Our research is a step towards achieving that goal.
